# Paraquat intoxication and associated pathological findings in three dogs in South Africa

**DOI:** 10.4102/jsava.v87i1.1352

**Published:** 2016-11-09

**Authors:** June H. Williams, Zandri Whitehead, Erna van Wilpe

**Affiliations:** 1 Department of Paraclinical Sciences, University of Pretoria, South Africa; 2 Department of Companion Animal Clinical Studies, University of Pretoria, South Africa; 3 Department of Anatomy and Physiology, University of Pretoria, South Africa

## Abstract

Paraquat is a bipyridylium non-selective contact herbicide commonly used worldwide. When ingestion occurs by humans and animals either accidentally, intentionally or maliciously, paraquat selectively accumulates in the lungs resulting in the production of oxygen-free radicals, causing membrane damage and cell death. Intoxicated subjects typically show progressive and fatal pulmonary haemorrhage, collapse and oedema. In individuals surviving the acute phase, pulmonary fibrosis develops. Gastrointestinal-, renal- and central nervous system clinical signs may also occur. Owing to the lack of effective treatment and absence of an antidote, the prognosis is poor. The clinical presentation, clinicopathological findings and treatment are briefly described of three dogs from one South African household, intoxicated with paraquat. Macroscopic and microscopic lesions in one dog that was necropsied, as well as pulmonary ultrastructure are detailed and illustrated for academic reference. All dogs presented with tachypnoea and dyspnoea 2–3 days after accidental paraquat ingestion. Treatment was aimed at reducing gastrointestinal absorption, enhancing elimination by diuresis and avoiding further oxidative damage by administration of antioxidants. All dogs, however, became progressively hypoxic despite treatment and were euthanised. Paraquat toxicity should be a differential diagnosis in dogs with unexplained progressive respiratory and gastrointestinal signs and renal failure. The local veterinary profession should be aware of accidental or intentional paraquat toxicity of animals. Existing literature, variations possible in canine clinical signs, measured parameters, lesions, as well as possible treatments, promising experimental antidotes and management options are discussed.

## Introduction

Paraquat (1,1’-dimethyl–4,4’-bypiridylium) is a non-selective, fast-acting bipyridylium herbicide active on foliage of all annual and certain perennial weeds. The liquid product Gramoxone^®^ (Syngenta Global, Schwarzwaldallee 215, Basel 4058, Switzerland) containing paraquat was only officially launched in 1982 although paraquat was in widespread use in Europe since 1958 (Copland, Kolin & Shulman 1974). Paraquat is also known as methyl viologen because of its dark blue–green colour (Dinis-Oliveira *et al*. 2008). Ingestion is the most common route of accidental or intentional exposure in humans and animals. Inhalation of aerosolised paraquat is not considered a hazard, causing only local nasal and tracheobronchial irritation according to several authors (Dinis-Oliveira *et al*. 2008; Krieger & Krieger 2001). However, the manufacturer’s information includes ingestion, inhalation and skin absorption as routes of fatal toxicity, stating also that the substance is very toxic to aquatic organisms and toxic to terrestrial vertebrates and invertebrates. Toxicity in humans via contact with damaged skin has been reported (Smith 1988).

Paraquat mainly affects the lungs, where it accumulates at up to 6–10 times the plasma concentration, sequestered in pulmonary type I, type II and Clara cells (Cope *et al*. 2004; Dinis-Oliveira *et al*. 2008; Krieger & Krieger 2001; Shuler *et al*. 2004). Oxygen-free radicals are formed resulting in acute alveolitis 1–3 days post-exposure. Tachypnoea, dyspnoea and cyanosis begin from 2 to 7 days post-exposure. If the affected animal or human survives, diffuse alveolar septal fibrosis and compensatory type II pneumocyte hyperplasia develop, followed by pulmonary fibrosis (chronic phase). Refractory hypoxaemia and eventual death occur from 5 days to several weeks later (Cope *et al*. 2004; Dinis-Oliveira *et al*. 2008; Gawarammana & Buckley 2011; Gfeller & Messonnier 1998).

Oropharyngeal and gastrointestinal inflammation, ulceration and sloughing are generally seen within the first few days after exposure (Cope *et al*. 2004). Signs referable to acute renal tubular necrosis may also occur (Chan *et al*. 1998; Shuler *et al*. 2004).

Radiographs taken in the acute phase may show the lungs to be normal or demonstrating an alveolar pattern, with progression to a diffuse coarse reticular nodular interstitial pattern (Dinis-Oliveira *et al*. 2008; Gfeller & Messonnier 1998; Shuler *et al*. 2004). Pulmonary ground-glass attenuation, bronchiectasis, parenchymal bands and subpleural interstitial thickening may be seen on thoracic computed tomography (Cope *et al*. 2004). Anaemia, leukocytosis, renal azotaemia, hypo- or hyperglycaemia, hyperlipasaemia, hyperlactataemia and hypokalaemia may be found on testing of whole blood and serum (Cope *et al*. 2004; Darke *et al*. 1977; Giri *et al*. 1983; Nagata 1992; Nagata *et al*. 1992 *et al*.; Takeshi, Talbot & Tomokazu 1989). Alkaline urine, proteinuria (Nagata *et al*. 1992) and mild systemic hypertension may also be present (Cope *et al*. 2004). Ante-mortem diagnosis is generally based on history of exposure, compatible clinical signs, plus toxicological analysis of vomitus, gastric content, bait/concentrate, urine and blood (Cope *et al*. 2004; Dinis-Oliveira *et al*. 2008; Gawarammana & Buckley 2011; Gfeller & Messonnier 1998).

On necropsy of affected dogs, the lungs typically appear heavy, dark and rubbery with haemorrhage and consolidation. Pneumomediastinum, mild serosanguinous hydrothorax and lesions of gastrointestinal irritation have also been reported. Microscopically, alveolar capillary congestion, oedema and collapse with over-distension of alveolar ducts and terminal bronchioles are observed in the acute phase, giving the lung a typical honeycomb appearance. Bronchiolar epithelial cell necrosis with desquamation into the lumen is also typical with minimal inflammatory cell response. Later, alveolar fibrosis and hyperplasia of bronchiolar epithelial cells are evident (Kelly *et al*. 1978).

No specific biochemical antagonist exists, although antioxidants such as acetylsalicylate, its lysine derivative and acetylcysteine are showing great promise experimentally in rats (Baltazar *et al*. 2013; Dinis-Oliveira *et al*. 2007, 2009; Gawarammana & Buckley 2011). Treatment aims to minimise toxicity by dosing of adsorbents such as activated charcoal, or Fuller’s earth (Gawarammana & Buckley 2011), administration of free radical scavengers and the institution of supportive care. Oxygen supplementation may increase tissue oxidative injury (Gawarammana & Buckley 2011; Shibamoto, Taylor & Parker 1990; Shuler *et al*. 2004) so should be avoided unless severe hypoxaemia develops. Regardless of treatment, the prognosis is poor.

## Case history

The owners of the dogs had bought Gramoxone 250^®^ (250 g/L of paraquat, Syngenta South Africa [Pty] Limited, Block 10, Thornhill Office Park 94 Bekker Street, Midrand, South Africa), available over the counter from the local farmer’s co-operative, in order to spray weeds in their plant nursery. Spraying of herbicides was usually performed by a responsible family member who used the spray judiciously only on identified weeds. On this particular occasion, a general nursery worker used the spray indiscriminately on several areas of the garden, and in so doing, the worker also contaminated the leftover maize-meal discarded from cooking pots in the area where the workers sat to eat. These scraps were regularly eaten by the three dogs without ill effect. The Gramoxone 250^®^ spray-soaked scraps were subsequently eaten by the dogs.

## Case presentation

The dogs, a three-year-old male castrated Pomeranian and two seven-year-old male castrated cross breeds, were presented to the Onderstepoort Veterinary Academic Hospital (OVAH) three days after ingestion of the paraquat. Acute vomiting of clear bright blue fluid with crumbly granules, corresponding with the colour of the herbicide and consistency of the cooked maize-meal, followed by inappetence, had been noted by the owner. The dogs were lethargic with congested mucous membranes, shortened capillary refill time, mild tachycardia, dehydration, severe ulcerative stomatitis and mild tachypnoea. Mild generalised muscle tremors occurred in one dog. All canine patients were normotensive, and abnormal lung sounds were not detected on thoracic auscultation. Peripheral blood oxygen saturation (SpO_2_) was above 95% at presentation in all dogs. Mild relative haemoconcentration and hyperalbuminaemia were present in two dogs. Moderate leukopaenia because of moderate neutropaenia and mild lymphopaenia, likely associated with endotoxaemia, were recorded in one dog. Hyponatraemia and hypokalaemia occurred in all dogs, likely secondary to vomiting and anorexia. One dog had mild type A hyperlactataemia (3.8 mmol/L [reference interval 0 mmol/L – 2.5 mmol/L]) that resolved post-hydration (1.4 mmol/L). Faecal evaluation negative for ova, alkaline urine (pH 8), mild proteinuria (1–2+/4) with inactive sediment and moderate glucosuria (2–3+/4) in the presence of normoglycaemia were found in all dogs. Thoracic radiographs showed no changes in one dog, mild perihilar broncho-interstitial lung pattern in another and diffuse reticular interstitial lung pattern in the third dog.

## Clinical management and outcome

Treatment was aimed at reducing paraquat absorption by administration of 1 g/kg activated charcoal (Medicolab CC, Randburg, South Africa) and 1 mL/4.5 kg lactulose (Lacson syrup 3.3 g lactulose/5 mL, Aspen Pharmacare, Sandton, South Africa) *per os* (PO) every 8 hours (h) (q8h) and metoclopramide 2 mg/kg/day intravenously (IV) (Clopamon 10 mg/2 mL injection, Aspen Pharmacare, Sandton, South Africa) at constant rate infusion (CRI). Elimination was enhanced by diuresis with IV crystalloid fluids (SABAX Ringer-Lactate/Hartmann’s Solution, Adcock Ingram Critical Care [Pty] Ltd., Johannesburg, South Africa), and further oxidative damage avoided by administration of antioxidants such as N-acetylcysteine 70 mg/kg PO q6h (Solumucol, Aspen Pharmacare, Sandton, South Africa) and vitamin E 300 IU PO q8h (Vitamin E capsules, Adminide Trading 17 cc, Sebenza, South Africa).

To reduce pulmonary inflammation and avoid fibrosis, prednisolone 0.5 mg/kg intramuscularly (IM) q24h (Prednisolone 1% Kela, Bayer Health Care Animal Health, Isando, South Africa) was administered. The dog presenting with muscle tremors was treated with diazepam 0.2 mg/kg IV bolus (Pharma-Q-Diazepam injection 10 mg/2 mL, Pharma-Q Holdings [Pty] Ltd., Johannesburg, South Africa) followed by methocarbamol 66 mg/kg PO q12h (Robaxin 500 mg tabs, Aspen Pharmacare, Sandton, South Africa) for 2 days. For the ulcerative stomatitis, 0.2% chlorhexidine gluconate (Orovet Oral Rinse, MEDPET, Johannesburg, South Africa) spray was applied PO q6h; ampicillin 20 mg/kg IV q8h (Ranamp 250, Ranbaxy SA, Centurion, South Africa) was administered for potential secondary bacterial infections and fentanyl 2 µg/kg/h IV CRI (Fresenius Kabi, Midrand, South Africa) for analgesia. Omeprazole 0.7 mg/kg PO q24h (Altosec, Aspen Pharmacare, Sandton, South Africa) and sucralfate 1 g/dog PO q8h (Ulsanic, Aspen Pharmacare, Sandton, South Africa) were given for possible gastrointestinal ulcers. Feeding was via naso-oesophageal intubation.

All dogs deteriorated over 1–6 days, showing signs of progressive tachypnoea and dyspnoea, hypoxaemia and increasing alveolar-arterial gas gradient (A-a gradient) compatible with diffusion impairment progressing to ventilation–perfusion mismatch. One dog became severely hypoxaemic (SpO_2_ < 90%, arterial partial pressure of oxygen [P_a_O_2_] 60 mmHg) requiring oxygen therapy and pressure-controlled mechanical ventilation (Engtröm Carestation, Datex Ohmeda Inc, Madison WI, USA) (Hopper & Powell 2013). As expected, no improvement was noted, and thus, euthanasia was performed. The remaining two dogs were also euthanased 2 and 6 days post-admission because of disease progression and grave prognosis.

## Macroscopic necropsy lesions

A necropsy was performed on one of the larger castrated mixed-breed male dogs approximately 12 h post-euthanasia after refrigeration of the carcass. Peripheral blood smear at the time of the necropsy showed leukocyte numbers apparently in normal range with mild active monocytosis, a few immature neutrophils, mild eosinophilia and small clumps of thrombocytes. Faecal flotation elicited a few *Ancylostoma caninum* ova. Urine pH was 6 and showed no abnormalities (Combur^9^Test, Roche Diagnostics GmbH, Sandhofer Strausse 116, D-68305 Mannheim, Germany).

**FIGURE 1:**
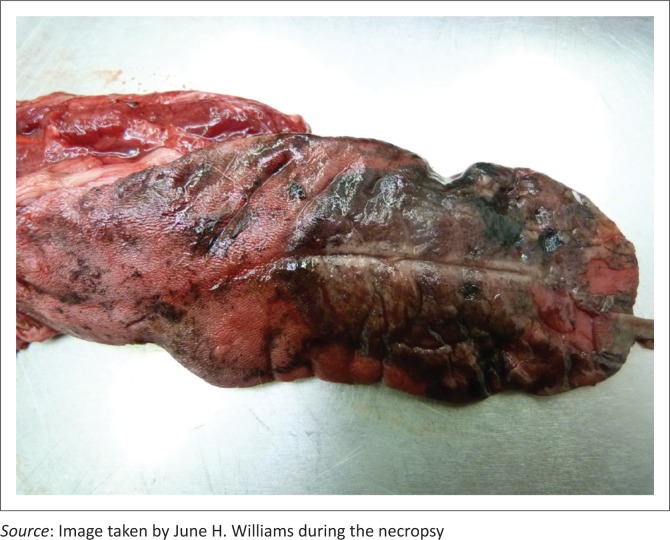
Tongue of paraquat-poisoned dog showing activated charcoal blackening, necrosis, dorsal tip epithelial sloughing, ulcerations and erosions.

**FIGURE 2:**
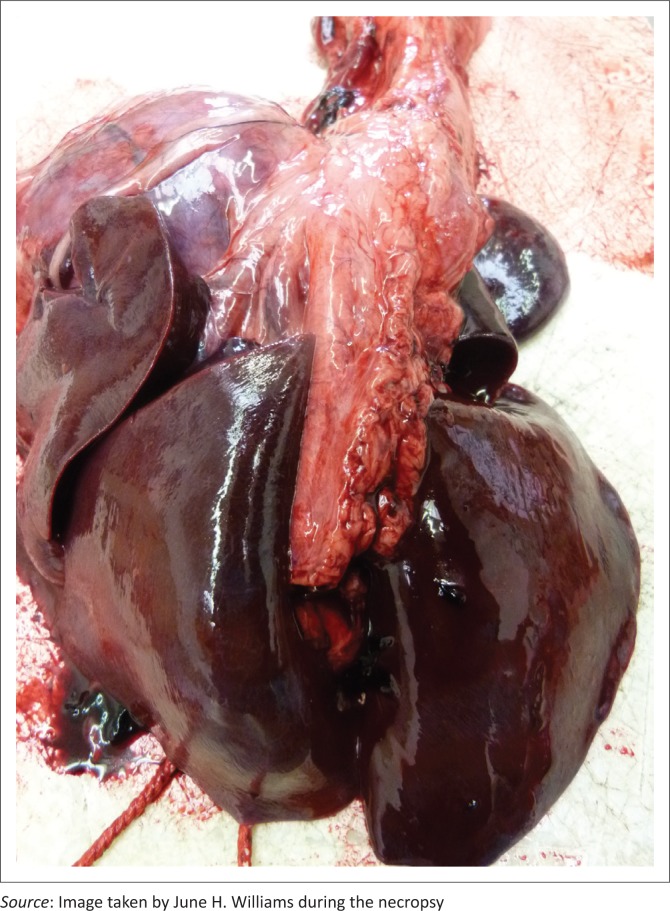
Dorsal view of dog lungs showing diffuse extreme dark congestion, oedema, lack of aeration and liver-like appearance: paraquat toxicity.

The dog was in good body condition. Fluid blackened with activated charcoal stained the mouth. Severe lingual erosions and ulcerations with epithelial sloughing and areas of necrosis were present involving the dorsal cranial half and tip of the tongue (Figure 1). There was moderate visceral congestion with splenomegaly, moderate serosanguinous hydropericardium and mild serosanguinous hydrothorax. The lungs were slightly smaller than normal, with a diffusely dark reddish-purple to black, solid, liver-like appearance, with increased consistency and wet on cut surface (Figure 2). Samples of all regions of the left lung sank completely in water and only some regions of the right lung partially floated, confirming atelectasis. There was some blood-tinged fluid lining the distal tracheal lumen.

The alimentary tract was almost devoid of food and faeces, with gastric mucosa blackened by activated charcoal, and the small intestine content was watery and bile-stained. Multifocal bilateral chronic renal scarring because of prior unrelated infarction and a large urine-filled cranial polar cyst present in the right kidney were incidental findings.

## Histopathology

Sections of 5 µm thickness prepared from alcohol-dehydrated, paraffin wax-embedded, 10% formaldehyde-fixed tissues from various organs were routinely stained with haematoxylin and eosin (HE) and examined by light microscope.

**FIGURE 3:**
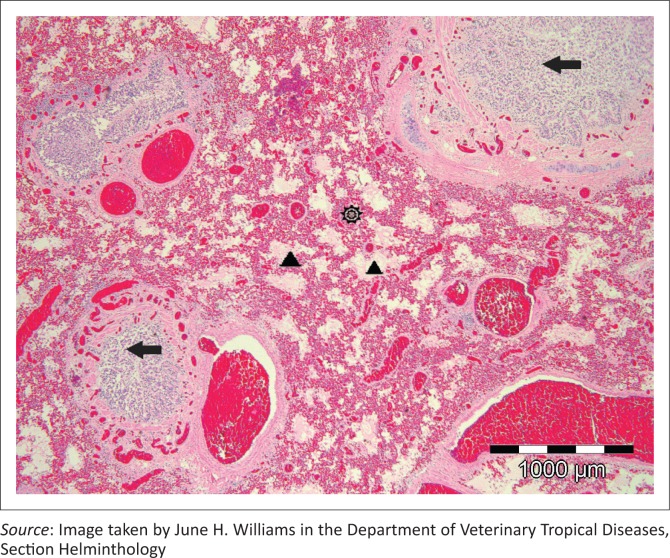
Low magnification of paraquat-induced extreme diffuse pulmonary congestion (star), high protein alveolar oedema (arrowheads) and honeycomb pattern of dilated respiratory bronchioles (arrowheads) and alveolar ducts with surrounding alveolar collapse (star) (HE x 40). Bronchiolar epithelium showing hyperplasia as well as degeneration, necrosis and desquamation into the bronchiolar lumens (horizontal arrows).

All areas of both lungs showed similar extreme diffuse vascular congestion, mild extravasation of erythrocytes into distal airways and alveolar ducts and marked distal airway protein-rich oedema, often fibrillary or forming fibrin/hyaline casts especially in respiratory bronchioles. A diffuse ‘honeycomb’ pattern was created by distended respiratory bronchioles and alveolar ducts surrounded by collapsed tortuous alveolar sac walls that lacked lining pneumocytes (Figure 3). There was an increase in alveolar macrophage numbers, unrecognisable cell debris and occasional sloughed degenerating epithelial cells scattered or clustered in the dilated oedema-filled spaces. All distal bronchi and bronchioles showed multifocal to diffuse intra-luminal sloughing of mucosal epithelial cells, these cells often filling the lumens (Figure 3). In some tertiary bronchi, the ciliated mucosa appeared hyperplastic and was thrown into folds projecting into the airway lumens. Some of the desquamated ciliated columnar cells remained attached to each other in short or wavy elongated strips, others were single and loose, and cellular changes varied from none on light microscopy to nuclear pycnosis and granular to disintegrating cytoplasm. Rare small clusters of bacterial rods and occasional neutrophils, often degenerating, were found scattered in bronchiolar lumens amongst the sloughed cells. A small amount of loose peribronchiolar lymphoid tissue with single cell lymphocyte necrosis was present as well as occasional mild anthrasilicosis.

The adrenal cortical *zona glomerulosa* had occasional small petechiae and single to few glandular epithelial cells showing degeneration or necrosis with a few neutrophils and lymphocytes present.

Mild acute nephrosis was evident with renal glomeruli having acute protein leakage into Bowman’s capsules, mild cortical tubular epithelial intracytoplasmic bile pigment and protein absorption, single epithelial cell necrosis and sloughing in tubular lumens of cortical and medullary rays and some globular protein casts in corticomedullary tubules. Chronic interstitial fibrosis with mild lymphoplasmacytic infiltration was found in the regions of unrelated chronic healed infarction.

Lingual epithelium in areas with gross lesions was necrotic and sloughed either in full thickness, leaving lingual papillae intact, or more commonly eroded and sloughed above the basal layer or deeper *stratum spinosum* cells. Some deep keratinocytes were oedematous. Small pustules of intact neutrophils and eosinophils were seen intermittently or in elongated bands intra-epithelially above the basal layer or subcorneally in areas of lingual epithelium adjacent to sloughed areas. Submucosally in affected regions of the tongue, oedema and mild perivascular infiltration of lymphocytes, some plasma cells and occasional eosinophils were seen, with eosinophilic leucostasis and fragmentation of erythrocytes evident in some blood vessels. Scattered submucosal mast cells had well-granulated cytoplasm.

The gastric mucosa was lined along the lumen with activated charcoal but without obvious lesions as were small and large intestine sections. The spleen was congested with some lymphocyte necrosis in mantle and marginal zones and had scattered red pulp haemosiderophages. Liver, heart and pancreas showed no significant lesions. In brain sections, oedema was seen in some areas.

## Ultrastructure

Formalin-fixed lung tissue samples were prepared for transmission electron microscopy by standard methods. Briefly, the samples were post-fixed in 1% osmium tetroxide in Millonig’s buffer, dehydrated through a graded ethanol series, infiltrated with a propylene oxide/epoxy resin mixture and embedded in absolute resin. Ultra-thin resin sections were stained with uranyl acetate and lead citrate and examined in a Philips CM 10 transmission electron microscope operated at 80 kV.

**FIGURE 4:**
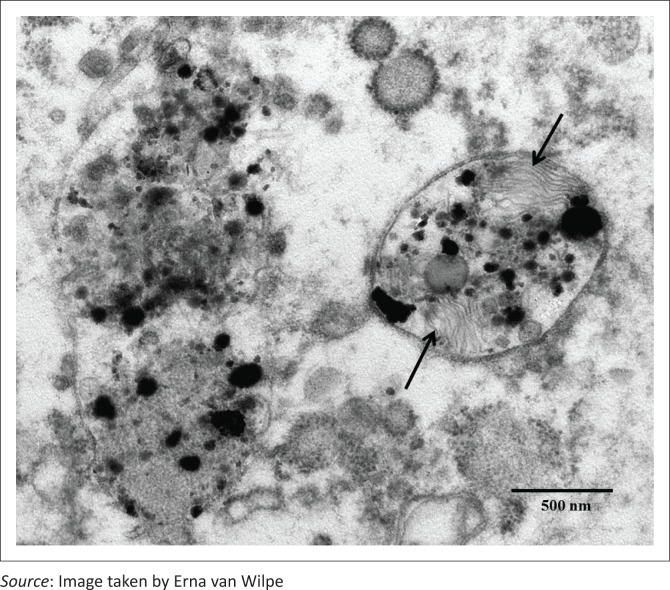
Interruption of the typical lamellar pattern (arrows) of the secretory vesicles of type II pneumocytes by the deposition of electron-dense material (electron microscopy/ EM).

**FIGURE 5:**
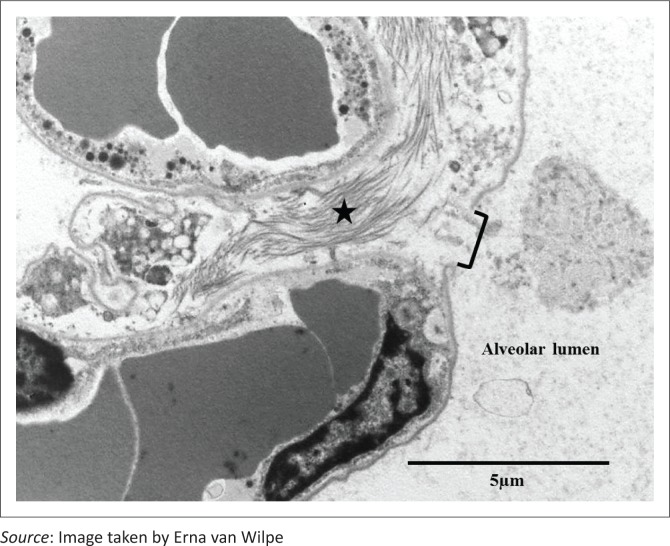
Ultrastructurally visible break (bracket) in a septal membrane with exposure of the interstitial tissue and collagen (star) underlying the alveolar lumen.

**FIGURE 6:**
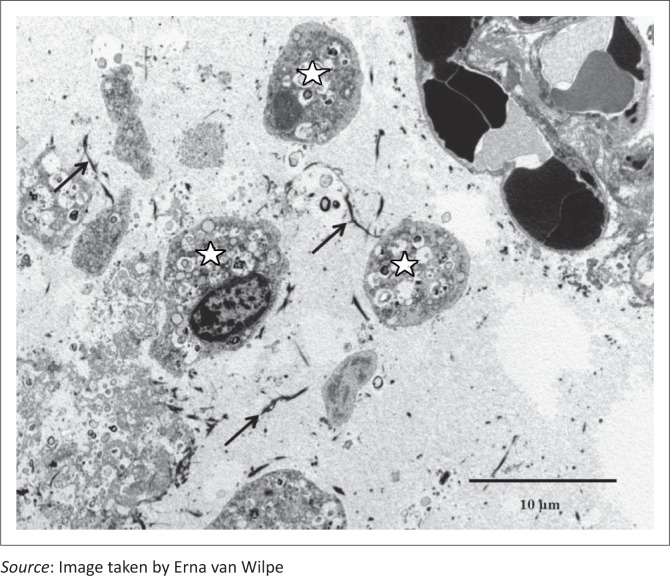
Fibrin deposits (arrows), desquamated pneumocytes (stars) and cellular debris in the alveolar lumen (EM).

**FIGURE 7:**
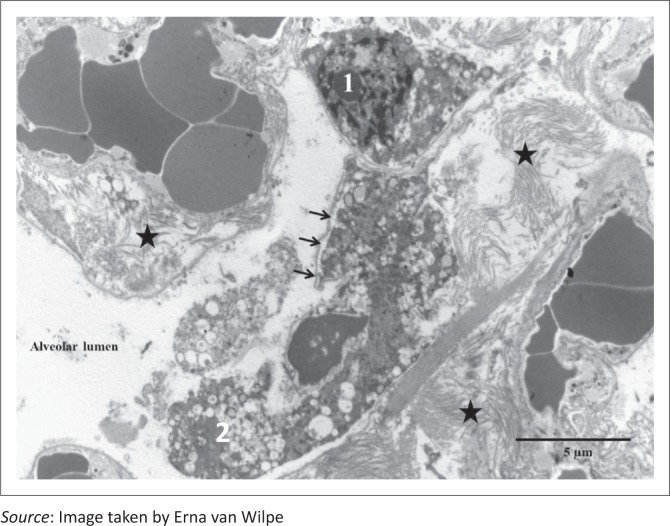
Fibroblast (1) within the alveolar septum and an intra-alveolar fibroblast (2) in an area of septal membrane (arrows) disruption. Note dilated endoplasmic reticulum in fibroblasts and interstitial collagen fibrils (stars) (EM).

The bronchioles revealed sloughing of ciliated epithelial cells with the underlying basement membranes being mostly intact and only a small number of breaks occurring. Alveolar collapse and loss of pneumocytes were apparent. The typical lamellar pattern of the secretory vesicles of type II pneumocytes was interrupted by the deposition of electron-dense material (Figure 4). The septal membranes displayed breaks in areas with exposure of the interstitial tissue underlying the alveolar lumina (Figure 5). Free red blood cells, fibrin deposits, desquamated pneumocytes and cellular debris were also present in the alveolar lumina (Figure 6). Fibroblasts with dilated endoplasmic reticulum as well as interstitial collagen fibrils were present within the alveolar septae (Figure 7). A few intra-alveolar fibroblasts were noted in areas where disruption of the septal membranes was present (Figure 7). The endothelial cells appeared to be active as evidenced by swelling and the presence of lysosomes.

The presence of eosinophils, with typical granules containing crystalline cores, was confirmed ultrastructurally amongst the neutrophils in the pustules of the tongue epithelium.

## Discussion

The blue–green vomitus and progressive clinical signs of the three dogs were typical of paraquat toxicity and considered diagnostic together with the owner’s history of purchasing the product and the subsequent indiscriminate use of it by an uninformed worker in their nursery. The colour and consistency of the dogs’ vomitus coincided with their ingestion of the maize-meal sprayed remnants left by the workers. Although various fresh tissues and alimentary tract content specimens were taken for confirmation of the toxin, no local laboratory could be found at the time that could analyse the specimens. However, all other evidence was considered conclusive of the diagnosis. In a previously reported series of seven maliciously paraquat-poisoned dogs, diagnosis of three was based on the typical history, clinical signs, lesions and progress (Cope *et al*. 2004). Paraquat ingestion resulting in high mortality and poor prognosis was witnessed in the three currently reported dogs, ascribed to its inherent toxicity and the lack of effective treatment. Reports of LD50 in dogs include 1.8 mg/kg (1.0–6.1) in male beagle dogs and 3.5 mg/kg (2.4–10.1) in female beagles administered once subcutaneously (Nagata *et al*. 1992) and 25 mg/kg – 50 mg/kg *per os* (Shuler *et al*. 2004). It was not possible to later estimate the dose ingested by these dogs, but it must have been in the orally toxic range. The herbicide was also sprayed indiscriminately over a large area of the garden, adversely affecting many plants. In order to reduce the consumed and retained paraquat dose, many paraquat products are formulated with emetics.

Although crackles have been reported (Gfeller & Messonnier 1998) on thoracic auscultation no abnormalities were found in these cases. They also demonstrated the thoracic radiographic variability as reported, with one dog having no radiographic changes and the other two dogs showing interstitial lung patterns as may be found in a more chronic stage.

All three canine patients were normotensive but mild systemic hypertension was reported (Cope *et al*. 2004). Clinicopathological findings were indicative of dehydration (haemoconcentration, mild hyperalbuminaemia and azotaemia in one dog). Neutropaenia because of paraquat toxicity, has been described in one human as well (Papanikolaou *et al*. 2001). Other described abnormalities such as hypo- and hyperglycaemia were not seen. Hypokalaemia may be ascribed to suddenly increased catecholamines, increased loss because of vomiting and also decreased intake. Lipase, cortisol, insulin, catecholamine and serum angiotensin I levels were not evaluated. Type B hyperlactataemia is typically described during paraquat toxicity because of the effect of oxygen-free radicals on mitochondrial function (Takeshi *et al*. 1989). In contrast, this study reports a type A hyperlactataemia because of the response to fluid therapy. Alkaline urine and proteinuria were seen as typical for paraquat poisoning.

As no proven antidote exists, attempts to prevent absorption of paraquat with oral mineral adsorbents such as Fuller’s earth or activated charcoal (Dinis-Oliveira *et al*. 2008; Gawarammana & Buckley 2011) preferably within 30–60 min of poison ingestion should be made although administration within 4 hours is still considered useful (Cope *et al*. 2004).

The mechanisms of paraquat toxicity include generation of free radicals and oxidative stress because of redox-cycling (Gawarammana & Buckley 2011). Paraquat is metabolised by several enzyme systems including NADPH-cytochrome P450 reductase, xanthine oxidase, NADH-ubiquinone oxidoreductase and nitric oxide synthase. In the presence of iron, hydroxyl-free radicals are formed, giving rationale to the use of desferoxamine (DFO) as a possible protective treatment: in mice and bacterial experiments with paraquat toxicity, DFO was protective, but in paraquat-poisoned rats there was no survival benefit with its use (Gawarammana & Buckley 2011). Nitric oxide (NO), which is produced from l-arginine by NO synthase, combines with superoxide to generate peroxinitrite, which is a potent oxidant and particularly toxic to lungs (Gawarammana & Buckley 2011; Moran *et al*. 2010).

Other key mechanisms of paraquat toxicity include lipid peroxidation of cell membranes, which compromises cell function and may trigger apoptosis; mitochondrial damage; oxidation of NADPH (decreased glutathione production); and activation of nuclear factor kappa B that binds in the nucleus to promoter regions to induce transcription of inflammatory mediators, which lead to platelet aggregation, fibrogenesis and attraction of inflammatory cells (Gawarammana & Buckley 2011). Apoptosis is induced by reactive oxygen species (ROS) and activation of nuclear factor kappa, as well as by peroxinitrite (Gawarammana & Buckley 2011). These multiple mechanisms likely explain why no single agent alters the course of intoxication substantially when given post-poisoning, barring salicylic acid, which, when administered at 200 mg per kg of sodium salicylate 2 h after rats were poisoned with 25 mg per kg of paraquat, resulted in 100% survival. The sodium salicylate treatment significantly reduced oxidative stress, nuclear factor kappa B activation, lipid peroxidation, platelet activation and reduced histological lung damage (Dinis-Oliveira *et al*. 2007). Lysine acetylsalicylate is a salicylate prodrug that can be administered parenterally, is available in a large number of human hospitals and is also effective at the same narrow dose range of 200 mg per kg in Wistar rats. Pulmonary collagen deposition was, however, observed histologically in animals that survived although it was less pronounced (Dinis-Oliveira *et al*. 2009). A subsequent experimental formulation of the standard Gramoxone (20% paraquat) with lysine acetylsalicylate dosed to adult Wistar rats by gavage resulted in full survival for 30 days at the highest dose tested (316 mg per kg lysine acetylsalicylate and 125 mg per kg paraquat) and the formulation also retained effective herbicidal activity (Baltazar *et al*. 2013).

Surfactant depletion and prevention of alveolar collapse may be corrected with ambroxol or exogenous surfactant (Dinis-Oliveira *et al*. 2008). Attempts may be made to decrease the extent of pulmonary inflammation and fibrosis with anti-inflammatory and immunosuppressant agents and collagen synthesis inhibitors, such as corticosteroids, chlorpromazine and cyclophosphamide, amongst others (Cope *et al*. 2004; Gawarammana & Buckley 2011), although controlled studies in humans have not been carried out and mortality rates remain high. Angiotensin type I converting enzyme inhibitors (Dinis-Oliveira *et al*. 2008) may also be useful as antifibrotic agents. Angiotensin II seems to act as a mitogen for fibroblast proliferation in lungs, linked to autocrine production of transforming growth factor β, a multifunctional cytokine that is the main cytokine involved in conversion of fibroblasts to myofibroblasts, with collagen synthesis, in the process of fibrosis (Ghazi-Khansari *et al*. 2007). The angiotensin type I converting enzyme inhibitors, enalapril and captopril, reduced paraquat-induced fibrosis in male albino Wistar rats, as shown by histopathology, and decreased hydroxyproline content of affected lung, without having any effect on glutathione and lipid peroxidation (Ghazi-Khansari *et al*. 2007).

Additional supportive care should be instituted including gastrointestinal protectants, analgesia, sedatives and tube feeding for adequate nutrition as required (Gawarammana & Buckley 2011; Shuler *et al*. 2004). Oxygen supplementation is not recommended (Johnson & Huxtable 1976; Shuler *et al*. 2004,) as it increases tissue oxidative injury by free radicals (Shibamoto *et al*. 1990). Taking the arterial blood gas values into account, oxygen therapy did worsen the clinical signs in the ventilated dog in this report. Retrospectively, a decision to euthanase should be made before ventilation is initiated unless the owner of the patient wishes to continue treatment despite the grave prognosis.

An additional new promising treatment is propofol for its scavenging properties (Dinis-Oliveira *et al*. 2008).

Within 12–24 hours of ingestion, 90% of absorbed paraquat is excreted unchanged in urine, with renal clearance decreasing rapidly with increasing time in severe poisoning due partly to increasing organ damage (Gawarammana & Buckley 2011). Diuresis is recommended to decrease the concentration of paraquat in the renal tubules to prevent kidney damage (Cope *et al*. 2004; Mizutani *et al*. 1992). Saline cathartics are helpful in fast removal of paraquat from the body (Krieger & Krieger 2001). Induction of vomiting or gastric lavage are contraindicated because of continued corrosive contact trauma to the gastrointestinal mucosa and have not been shown to be of value in the treatment of oral paraquat poisoning in humans (Gawarammana & Buckley 2011; Mizutani *et al*. 1992). Extracorporeal elimination enhancement by haemoperfusion and haemodialysis are alternatives with limited or questionable benefit (Gawarammana & Buckley 2011). Paraquat is actively taken up against a concentration gradient into type II pneumocytes with very slow elimination from this compartment (Gawarammana & Buckley 2011).

Prevention of pulmonary damage may be attempted by detoxifying reactive oxygen species by administration of other antioxidants such as superoxide dismutase, vitamin E, vitamin C, N-acetylcysteine, salicylic acid and fatty acids, amongst others, with variable results (Cope *et al*. 2004; Dinis-Oliveira *et al*. 2008; Gawarammana & Buckley 2011) by reducing redox-cycling with methylene blue and by reducing paraquat lung accumulation, although all *in vivo* studies have failed to show consistent positive results. Putrescine, D-propanolol and imipramine have only shown good effects *in vitro* and anti-paraquat antibodies or anti-paraquat antigen-binding fragments resulted in the sequestration of the toxin in the plasma compartment but did not prevent paraquat accumulation in the lungs (Bowles *et al* 1992; Dinis-Oliveira *et al*. 2008; Gwaltney-Brant & Rumbeiha 2002).

Based on gross and microscopic pathology of paraquat toxicity as previously described in 10 dogs (Kelly *et al*. 1978), the current case was classified on pulmonary pathology as representing the acute stage of extreme congestion, haemorrhage and oedema at the time of euthanasia. Acute cases survive up to 2–3 days (Caswell & Williams 2007). Histological lesions concurred with those previously described at this stage including the honeycombing, paucity of inflammatory leukocyte exudation, presence of fibrin and variable epithelial hyperplasia, necrosis and desquamation of bronchiolar epithelium into the airways (Kelly *et al*. 1978). In the review by Kelly *et al*. (1978), the loss of type I and II pneumocytes was mentioned and shown ultrastructurally, and irrespective of the stage of the toxicity, an abundant increase in alveolar fibroblasts on either side of the basement membrane was seen. In the present case, some fibroblasts were noted in the septal walls as well as within alveoli where basement membranes were damaged.

The osmiophilic granular structures mentioned within the alveolar lumina (Kelly *et al*. 1978) were absent in this case. Active endothelial cells were demonstrated in the present case, in contrast to the findings by Kelly *et al*. (1978) of normal endothelium in the alveolar capillaries.

Loss of especially type II pneumocytes results in reduced surfactant synthesis and secretion, leading to increased intra-alveolar surface tension, alveolar oedema and collapse. This is reported to be the primary lung lesion during the early stages of paraquat toxicity in humans and experimental rats, and in rats, this occurs as early as 3 days post-administration of paraquat (Kelly *et al*. 1978; Smith & Heath 1975). In the current case, ultrastructurally the typical lamellar pattern of the secretory vesicles of type II pneumocytes was interrupted by the deposition of electron-dense material. The significance of this finding is uncertain (Figure 4). Despite an extensive literature search covering ultrastructural post mortal and similar pathological changes in humans and animals, no reference could be found that described similar secretory vesicle changes. Lamellar bodies are members of the secretory lysosome subclass of lysosome-related organelles (Weaver, Na & Stahlman 2002). Surfactant phospholipids are the principal constituents of lamellar bodies and are organised into tightly packed bilayer membranes, strongly influenced by peptide SP-B, which is lung-specific and hydrophobic (Weaver *et al*. 2002). Smith and Heath (1975) showed in rats injected with paraquat intra-peritoneally that within 8 hours post-poisoning, type I pneumocytes showed vacuolation and disruption of organelles, and after 18 hours, type II pneumocytes showed swelling of mitochondria and vacuolation of lamellar bodies with disruption of endoplasmic reticulum; by day 2, both type I and II pneumocytes started to disintegrate, and by day 3, alveolar walls were denuded of epithelial cells.

Pulmonary haemorrhage and oedema were thought to be as a result of increased permeability of capillary endothelial cells (Kelly *et al*. 1978) despite lack of obvious damage to pulmonary capillaries (Smith & Heath 1975). Smith and Heath (1975) postulated that increase in vascular permeability is likely related to loss of surfactant production by type II pneumocytes, leading to increased surface tension of alveolar fluid, which also would result in hyaline membrane production.

In the current case, although some desquamation could have been ascribed to autolysis, the bronchial and bronchiolar epithelial cells themselves were not showing autolytic change, and the resulting obstruction of these airways must also have contributed to the severe and progressive clinical dyspnoea.

Paraquat is a vesicant and the ulcerative stomatitis, and damage to the alimentary tract seen in toxicity cases is caused by its corrosive effect (Smith & Heath 1975; Volmer 2008). In the review by Kelly *et al*. (1978), only 1 of 10 dogs was reported to show alimentary tract irritation but this manifested as pharyngeal epithelial necrosis and ulceration with underlying inflammation, as well as gastric submucosal oedema, lymphatic dilation and mild inflammation. The significance of eosinophils amongst the neutrophils present in lingual epithelial pustules in the current case is uncertain.

Renal lesions similar to the present case were described in most of the dogs in the study by Kelly *et al*. (1978), with severe tubular degeneration occurring in two dogs that had increased blood urea nitrogen levels. Paraquat-associated renal tubular damage has been described in many species including rats, mice, guinea pigs, monkeys and man and may result in anuric renal failure (Kelly *et al*. 1978). In the current case, the renal changes at necropsy appeared early and acute in kidneys that had healed from previous unrelated chronic injury, and urinalysis during the necropsy did not yet reflect the acute injury.

Adrenocortical necrosis with mild associated inflammation, involving individual or groups of cells in the *zona glomerulosa,* was seen in the current case and in 5 of the 10 dogs studied by Kelly *et al*. (1978). This lesion is only described in experimental paraquat toxicity in mice and is not commonly recognised in other species. Paraquat is chemically similar to 1,1 dichloro-2-(o-chlorophenyl)-2-(p-chlorophenyl)ethane (o,pDDD), which is used therapeutically for that action in the treatment of hyperadrenocorticism, except that the *zona fasciculata-reticularis* is affected and not the *zona glomerulosa* (Kelly *et al*. 1978). The extent of this lesion was ascribed to the dose of paraquat received because the dog with the worst adrenocortical necrosis had rapid fatal illness lasting less than 24 hours (Kelly *et al*. 1978).

Multifocal myocardial necrosis was not present in the current case but Kelly *et al*. (1978) reported it in 4 of 10 cases, and in 2 of those dogs small and large intramural and epicardial arterioles/arteries displayed segmental or diffuse mural fibrinoid necrosis. These appeared to be unique lesions in the dogs and may have resulted from altered coronary perfusion either by thrombosis (which was not seen in that series) or vasospasm. Electrocardiographic abnormalities associated with paraquat toxicity have been seen in humans but were not substantiated by histopathology (Kelly *et al*. 1978).

In one experimental study in 18 beagle dogs in 3 groups of 6 injected subcutaneously with 3 different doses of paraquat, no electrocardiographic abnormalities were detected (Nagata 1992). In that study, slight hepatocyte degeneration and swelling, focal necrosis, slight congestion with mild haemorrhage bordering the gallbladder and mild gallbladder mucosal papillary hyperplasia were found. In another study in which beagles were injected with higher doses of paraquat (Nagata *et al*. 1992), dogs died of respiratory distress and renal failure, with surviving animals showing pulmonary fibrosis, alveolar type II pneumocyte proliferation, renal calcium deposition, thickening of basement membranes and proximal renal tubular epithelial necrosis. In a report of malicious paraquat poisoning of dogs in Oklahoma, USA, pulmonary and renal lesions were observed in five dogs (Bischoff *et al*. 1998).

Only one of the three dogs in the current report showed neurological signs in the form of generalised muscle tremors, attributed to brain oedema that was confirmed on necropsy. Other neurological signs reported include seizures, hyperexcitability and incoordination (Gfeller & Messonnier 1998).

## Ethical considerations

This is, in part, a retrospective case report, describing three clinical cases of client-owned pets using data retrieved from the patient record system of the OVAH. The animals reported, while alive, were treated and housed according to the standard OVAH protocols for the management of client-owned pets. All diagnostic tests were performed as part of the routine workup. No diagnostic tests or treatments were conducted for research purposes. The owner gave permission and encouraged the use of these cases for publication in order to enlighten veterinarians and other animal owners of the dangers of paraquat use and toxicity.

## Conclusion

Paraquat toxicity should be considered a differential diagnosis in dogs with unexplained respiratory and gastrointestinal signs, renal disease (Shuler *et al*. 2004), and a history of paraquat presence or environmental use. Owing to the ease with which the herbicide Gramoxone 250^®^ (Syngenta) may be purchased in South Africa, paraquat toxicity is a potential toxicity in humans and animals, either accidental, intentional (as in suicide) or with malicious intent. The lack of a proven antidote in humans and animals and the progression of severe pulmonary lesions with fibrosis make treatment and management of cases challenging and with a generally poor prognosis. High-dose parenteral lysine acetylsalicylate, various other related and non-related antioxidants and other substances, such as some angiotensin type I converting enzyme inhibitors, however, have shown great promise as early treatments and for prevention of progressive fibrosis in experimental studies in rats.
